# Advances in the application of recombinant herpesvirus-vectored vaccines in animal disease control

**DOI:** 10.3389/fmicb.2026.1888296

**Published:** 2026-07-21

**Authors:** Jiahui Guo, Chen Mei, Xinyao Sun, Qiuhui Lin, Weidong Bu, Shijie Xie, Bo Jiang

**Affiliations:** 1 Beijing Key Laboratory for Prevention and Control of Infectious Diseases in Livestock and Poultry, Beijing, China; 2 Institute of Animal Husbandry and Veterinary Medicine, Beijing Academy of Agriculture and Forestry Sciences, Beijing, China; 3 The Platform of Youth Innovation, Institute of Animal Husbandry and Veterinary Medicine, Beijing Academy of Agricultural and Forestry Sciences, Beijing, China; 4 College of Life Sciences and Food Engineering, Hebei University of Engineering, Handan, Hebei, China; 5 College of Life Sciences, Henan Normal University, Xinxiang, Henan, China

**Keywords:** cat, herpesvirus, immune response, pig, poultry, recombinant vaccine

## Abstract

Recombinant herpesvirus vectors have emerged as highly potent platforms for next-generation vaccine development, distinguished by their genomic capacity and their unique ability to elicit durable, long-lasting immunity through persistent latent infections. In this review, we trace the evolutionary trajectory of herpesvirus vector engineering—transitioning from traditional homologous recombination to advanced bacterial artificial chromosome (BAC) systems and precise, scarless CRISPR/Cas9 gene editing. We comprehensively evaluate the rational design of classical animal herpesvirus vectors, including pseudorabies virus (PRV), herpesvirus of turkeys (HVT), and feline herpesvirus type 1 (FHV-1). Specifically, we highlight their distinct advantages in molecular attenuation, the optimization of non-essential insertion loci, and the application of tissue-specific promoters for multiplexed antigen presentation. Furthermore, we discuss the strategic deployment of multivalent herpesvirus vaccines within the “One Health” framework, emphasizing their critical role in simplifying immunization protocols and interrupting the transmission chains of zoonotic diseases. Finally, we address the prevailing translational bottlenecks, including scalable manufacturing challenges and stringent regulatory frameworks regarding environmental release, providing perspectives on how continuous biotechnological innovations will empower herpesvirus vectors to serve as formidable prophylactic tools against emerging and re-emerging infectious diseases.

## 1 Historical development of diverse herpesvirus vectors

Herpesviruses possess large and structurally complex double-stranded DNA (dsDNA) genomes, typically ranging from 120 to 250 kb (Bruckner et al., 1991; Gruffat et al., 2016; Ibanez et al., 2018; Marintcheva and Weller, 2003; Martinez-Guzman et al., 2003). This substantial genomic capacity confers a pronounced inherent advantage for their application as recombinant vaccine delivery vectors. Early investigations revealed the presence of numerous genetic loci within the herpesvirus genome that are dispensable for viral replication *in vitro*. Prominent examples include the gE/gI, TK, and specific non-essential UL regions of pseudorabies virus (PRV) (Wang et al., 2023); the meq and pp38 loci of Marek’s disease virus (MDV) (Sun A. et al., 2022); and the TK and gG regions of feline herpesvirus type 1 (FHV-1) (Tang et al., 2023; Yang et al., 2023). The targeted deletion of these regions not only effectively attenuates viral virulence—thereby ensuring the safety profiles of the resultant vaccines—but also provides ideal insertion sites capable of accommodating the chimeric expression of large or multiple foreign antigenic cassettes. Furthermore, the capacity of herpesviruses to establish hallmark latent infections within the host facilitates the continuous stimulation of the immune system, thereby eliciting robust and highly durable humoral and cellular immune responses (Giri and Batra, 2025; Zhu et al., 2024). Capitalizing on these advantages, researchers have screened and engineered a diverse array of specialized herpesvirus vector systems, tailored to address specific host pathogens and the pragmatic requirements of clinical disease control.

**FIGURE 1:**
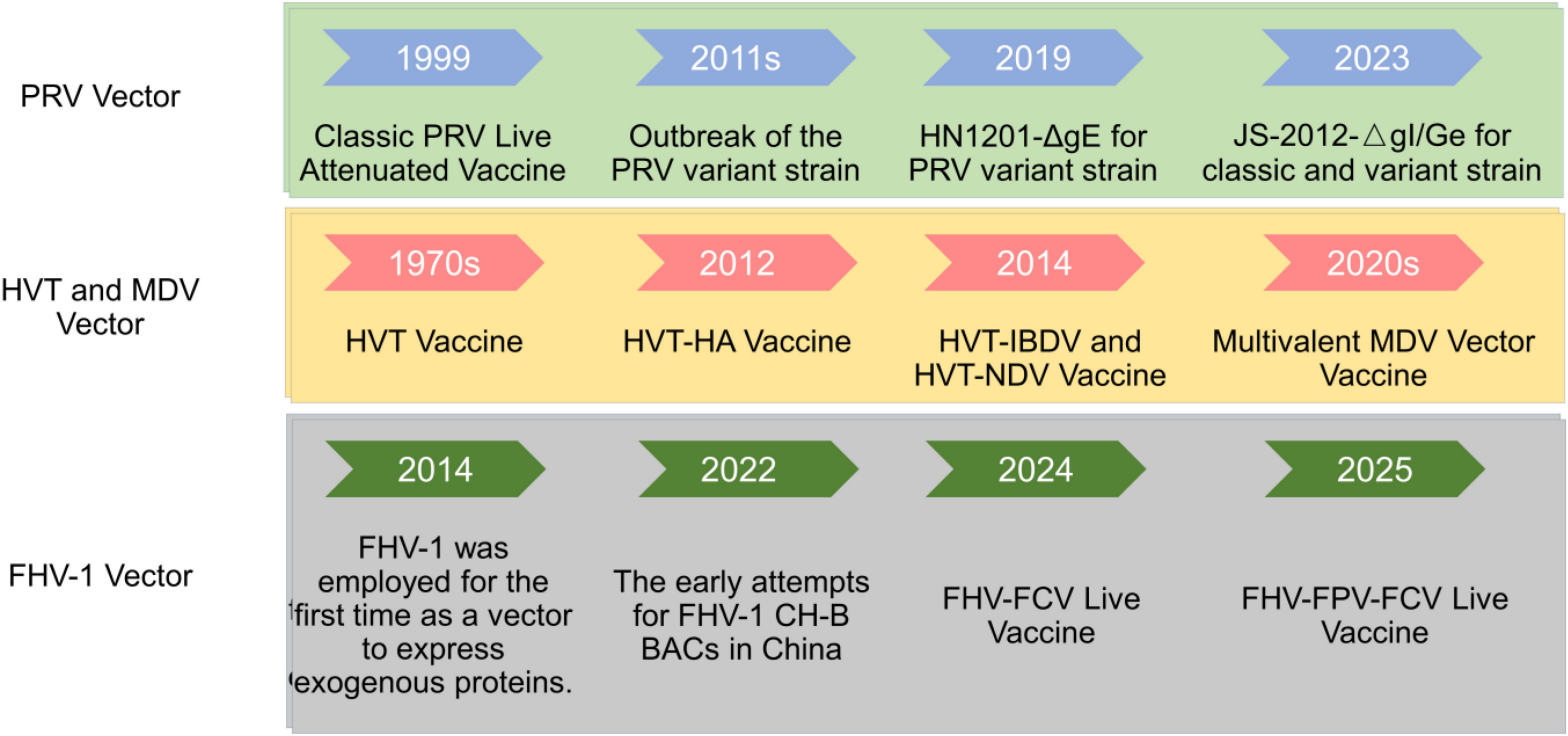
Timeline of key developments in animal herpesvirus vaccines. In this figure illustrates the evolution of three distinct vaccine platforms: the Pseudorabies Virus (PRV) vaccine, which progressed from classic strains to variant-specific vaccines; the Avian Herpesvirus (HVT and MDV) platform, which advanced from monovalent to multivalent viral vectors; and the Feline Herpesvirus (FHV-1) platform, recently developed into multivalent live vaccines (shown here are representative viral vectors). The timeline highlights the overall trend from conventional vaccines to complex recombinant vector technologies in the field of animal herpesvirus vaccines.

Within the Alphaherpesvirinae subfamily, herpes simplex virus (HSV) and varicella-zoster virus (VZV) are predominantly utilized in human medicine (Ansari et al., 2021; Bertelli et al., 2023; Bo and Li, 2022; Cui et al., 2024; Dohner et al., 2024; Hakami et al., 2024; Jones, 2019; Liu et al., 2022; Madavaraju et al., 2020; Nie et al., 2023; Ostler and Jones, 2023; Pan et al., 2023; Verzosa et al., 2021; Wu et al., 2020; Zhao J. et al., 2020). Notably, owing to its pronounced neurotropism, broad host cell tropism, and thoroughly characterized genomic background, HSV has not only secured a pivotal role in the development of vaccines against human infectious diseases but has also demonstrated substantial translational potential in targeted oncolytic virotherapy for malignancies in recent years (Andtbacka et al., 2015). In the veterinary domain, PRV, serving as a classical model for animal herpesviruses, has emerged as an optimal vector for the construction of multivalent recombinant swine vaccines (e.g., bivalent or trivalent vaccines against classical swine fever and porcine circovirus). This is largely attributable to its ability to induce potent immune responses in swine populations and the availability of well-established, safe attenuated strains (such as Bartha-K61) (Delva et al., 2020; Figure 1).

For the prevention and control of avian infectious diseases, vector systems predicated on the herpesvirus of turkeys (HVT) and MDV have achieved remarkable commercial success on a global scale. HVT, in particular, exhibits negligible pathogenicity in poultry, effectively circumvents the inhibitory effects of maternally derived antibodies, and confers lifelong protection following a single in ovo or subcutaneous inoculation. Consequently, it is widely regarded as the gold-standard vector for the expression of antigens from the Newcastle disease virus (NDV) and the infectious bursal disease virus (IBDV) (Calderon et al., 2022; Criado et al., 2023; Zhang et al., 2024). Additionally, feline herpesvirus type 1 (FHV-1) has garnered considerable attention for the development of mucosal vaccines targeting the upper respiratory tract of companion animals, a consequence of its robust tropism for the respiratory mucosa and conjunctiva of felines (Lee et al., 2021; Tian et al., 2025).

In summary, the fundamental advantages of herpesvirus vectors extend beyond their safety and tissue-specific tropism within the target host; they fundamentally encompass their intrinsic capacity to elicit potent immunogenicity, the extensive carrying capacity afforded by non-essential genetic loci, and their highly reliable genetic stability during large-scale manufacturing.

## 2 Historical development of construction strategies for recombinant herpesvirus vaccines

Propelled by rapid innovations in molecular biology, the technologies for constructing and modifying herpesvirus vectors have undergone several milestone transitions, which have significantly accelerated the translation of recombinant vaccines from basic research to clinical application. During the initial exploratory phase, vector construction relied predominantly on traditional intracellular homologous recombination (Tan et al., 2022). This approach typically involves the use of restriction endonucleases to generate transfer plasmids containing foreign genes flanked by homologous sequences. These plasmids are co-transfected with the full-length viral genome into permissive cells, where recombinant viruses are generated via the host cell’s endogenous DNA homologous recombination repair machinery. Characterized by high precision and genetic stability without the need for exogenous enzyme systems, this technique has been widely applied in the construction of various animal herpesvirus vaccine vectors. For instance, Zhao et al. successfully generated the PRV Fa TK-deleted strain by co-transfecting the genome of the PRV Ea TK− mutant with the TK-deletion plasmid pUCPB (Zhao J. et al., 2020). Although this technique established the methodological foundation for vector construction, its extremely low recombination frequency (typically <1%) necessitates multiple rounds of plaque purification using fluorescent proteins or drug-resistance genes as selectable markers. Consequently, this results in protracted construction cycles and impedes complex, multiplex gene editing.

To overcome the bottleneck associated with the *in vitro* manipulation of large viral genomes, bacterial artificial chromosome (BAC)-based reverse genetics emerged and rapidly became the predominant core tool in the field (Wussow et al., 2009). By cloning the entire herpesvirus genome into *Escherichia coli* plasmids containing specific replicons, researchers enabled the direct modification of massive viral genomes within bacteria using the highly efficient Red/ET recombination system. The introduction of the BAC system completely bypassed the low recombination efficiency inherent to eukaryotic cells, facilitating the stable maintenance and complex sequence modification of viral genomes within prokaryotes. For example, Jons et al. utilized the BAC system to construct a recombinant PRV expressing GFP and containing LoxP sites (Jons and Mettenleiter, 1997). Furthermore, researchers established reverse genetics systems for the PRV variant strains HLJ8 and LA2017 based on the BAC platform in 2016 and 2017, respectively, providing novel tools for vaccine development (Tian et al., 2025). The successful application of the PRV BAC system has allowed researchers to rapidly knock out multiple virulence-associated genes and tandemly insert expression cassettes from different pathogens, thereby significantly accelerating the iterative development of novel multivalent marker vaccines for swine. In addition, Shah et al. employed BAC technology to construct recombinant HVT vaccines (vHVT-AI, vHVT-IBD-AI, and vHVT-ND-AI) expressing the AIV COBRA H5, IBDV VP2, and NDV F proteins, respectively (Shah et al., 2022; Zai et al., 2022). Challenge assays demonstrated their robust protective efficacy and viral shedding control. Shah et al. inserted the VP2 expression cassette of the IBDV NJ09 strain into the HVT UL55-UL56 non-coding region to generate the recombinant virus HVT-VP2-09, which exhibited growth characteristics similar to the parental strain in cell culture and conferred effective immune protection against virulent challenge (Shah et al., 2022). Regarding feline herpesvirus research, Costes and Tai constructed FHV-1 BAC clones using the gG gene and the region upstream of UL56 as insertion sites in 2006 and 2010, respectively, laying the groundwork for FHV-1 vector vaccine development (Tai et al., 2010). However, the BAC system also possesses certain limitations. These include the susceptibility to genomic fragment loss during electroporation, complicated operational procedures, and heavy reliance on bacterial culture systems. Moreover, the introduction of bacterial regulatory sequences may interfere with the normal replication and rescue of the virus.

In recent years, the rapid development and widespread application of CRISPR/Cas9 gene-editing technology have ushered in a new era of enhanced precision and efficiency for herpesvirus vector engineering (Asmamaw and Zawdie, 2021). By utilizing guide RNAs to direct the Cas9 nuclease to induce targeted double-strand DNA breaks at specific loci within the viral genome, the CRISPR/Cas9 system not only increases recombination or insertion efficiencies by several orders of magnitude but, more importantly, precisely mediates homology-directed repair (HDR) or non-homologous end joining (NHEJ). For instance, using the PRV HB2017 strain as the parent, Zhang et al. employed the CRISPR/Cas9 system to simultaneously knock out the TK, gE, and gI genes, yielding the attenuated strain PRV HB2017ΔTKΔgE/gI, which exhibited excellent performance in terms of cell proliferation, genetic stability, safety, and immunogenicity (Zhang Huawei et al., 2020). Furthermore, in the development of HVT vector vaccines, the integration of CRISPR/Cas9 technology has not only drastically reduced the construction cycle of candidate vaccines from months to weeks but has also enabled genuine “scarless editing” (Apinda et al., 2023; Calderon et al., 2022; Zhang et al., 2024). In the context of feline herpesvirus, Tang et al. established the first CRISPR/Cas9-mediated FHV-1 recombination system, achieving the targeted knockout of virulence genes (such as gI/gE and TK) and the insertion of antigens, thereby obtaining a candidate vaccine strain that is safe, effective, and capable of inducing significant immune responses in cats (Tang et al., 2024; Tang et al., 2023). Such scarless manipulation effectively averts the potential biosafety risks associated with the retention of exogenous marker genes, aligning with the stringent regulatory standards and clinical safety requirements for modern veterinary and human biological products. Despite the pronounced advantages of CRISPR/Cas9 technology, issues such as potential off-target effects and incomplete editing still necessitate systematic optimization to enhance its application reliability. From a molecular perspective, “incomplete editing” primarily manifests as competition between inefficient homology-directed repair (HDR) and the dominant non-homologous end-joining (NHEJ) pathway in host cells. This competition often leads to random and unintended insertion or deletion (indel) mutations at the cleavage sites, resulting in mosaicism within the recombinant viral population or loss-of-function mutations in essential genes. To overcome these technical limitations, current optimization strategies are shifting toward the use of high-fidelity Cas9 nuclease variants (such as HiFi Cas9) to minimize off-target rates, and the adoption of transient ribonucleoprotein (RNP) complex delivery to shorten the cleavage window. Furthermore, these approaches are being combined with small-molecule pathway modulators to selectively enhance the efficiency of homology-directed repair (HDR), thereby ensuring the genetic purity of the recombinant viral strains and their absolute safety for clinical applications. Beyond the aforementioned technologies, researchers have also successfully applied Fosmid library technology to the vaccine development of vectors such as HVT and PRV (Qi et al., 2020; Zhou et al., 2018).

**FIGURE 2:**
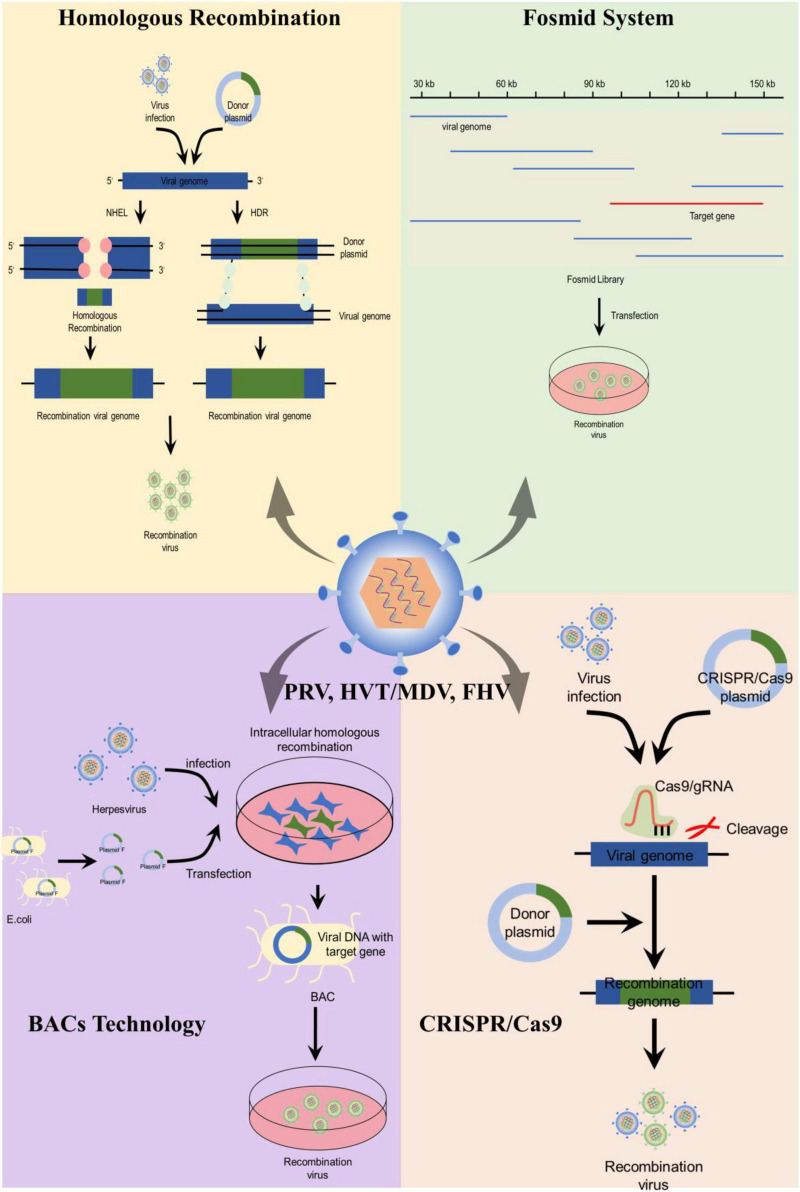
Schematic illustration of four principal genomic engineering strategies for constructing recombinant herpesvirus vaccines. In this figure compares the mechanisms and workflows of four key technologies used in herpesvirus vector development: (Top Left) A classical method based on co-transfection of viral genome and a donor plasmid carrying the gene of interest (GOI) with homologous arms. The GOI is integrated into the viral genome via cellular homologous recombination or HDR, yielding recombinant virus. This process is often inefficient and requires laborious screening. (Top Right) Used for large viral genomes, the genome is split into overlapping fosmid clones maintained in *E. coli*. One clone carries the desired modification. Co-transfection of the full fosmid set into eukaryotic cells allows reconstitution of the full-length modified viral genome via homologous recombination, generating recombinant virus. (Bottom Left) The entire viral genome is maintained as a BAC in *E. coli*. Precise genetic modifications are efficiently introduced using bacterial genetics. Transfection of the modified BAC into mammalian cells rescues the recombinant virus. (Bottom Right) A modern editing platform where a gRNA directs Cas9 to cleave a specific viral genomic site. A donor plasmid supplied during infection enables precise GOI insertion via cellular repair mechanisms. This approach greatly enhances the speed and efficiency of recombinant virus generation.

The developmental trajectory of herpesvirus vector platforms represents an evolutionary history transitioning from the exploitation of biological characteristics to profound empowerment by modern genetic engineering (Figure 2 and Table 1). Regarding the selection of diverse vectors, the research paradigm has shifted from early empirical explorations reliant solely on naturally attenuated strains to a rational design phase predicated on specific tissue tropism, genomic capacity, and host-pathogen immune interactions. On the technological front of vector engineering, the continuous circumvention of technical barriers—progressing from time-consuming and inefficient traditional homologous recombination, through the macroscopic and stable whole-genome manipulation reliant on BAC systems, to the microscopic, scarless, and precise editing of the CRISPR/Cas9 era—has not only endowed herpesvirus vectors with safety profiles and multivalent loading potentials but also established a robust methodological foundation for the rapid response to emerging and re-emerging infectious diseases in the future.

**TABLE 1:** Advantages and disadvantages of recombinant herpesvirus construction methods.

Technical method	Advantages	Disadvantages
Homologous recombination	1. High precision, good genetic stability.	1. Low recombination efficiency, time-consuming screening.
	2. Does not rely on exogenous enzymes, relatively traditional operation.	2. Complicated procedure.
	3. Mature technology, widely applied.	3. Insertion site may affect virus replication and immune properties.
Fosmid library technology	1. Efficiently clones large genome fragments.	1. Limited by virus genome operation and screening.
	2. Simpler operation compared to BAC technology.	2. Cloning efficiency is affected by plasmid stability and transfection efficiency.
	3. Suitable for integrating multiple exogenous genes.	3. May have limitations in large-scale applications.
Bacterial artificial chromosome (BAC) technology	1. Can handle large and complex viral genomes.	1. Complex operation, requires strict bacterial culture and screening systems.
	2. High efficiency, low cost, good precision in bacterial systems.	2. Virus genome may lose fragments during electroporation.
	3. Simplifies recombinant virus construction.	3. Bacterial sequences introduced may interfere with virus replication.
		4. The genetic instability caused by the spontaneous excision or retention of the mini-F replicon.
		5. The potential for unintended homologous recombination
CRISPR/Cas9 gene editing	1. Efficient, precise, and fast.	1. Potential off-target effects.
	2. Strong specificity, powerful editing ability.	2. Incomplete gene editing may occur.
	3. Wide applicability, can be used for various viral vectors.	3. The technology system still needs optimization to ensure stability.

## 3 Advantages and characteristics of animal herpesvirus vectors

**FIGURE 3:**
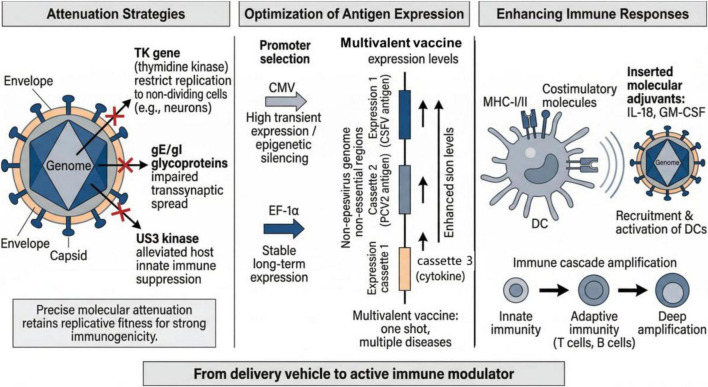
Rational design of recombinant herpesvirus vectors. Attenuation Strategies (Left): Targeted deletion of specific virulence genes (e.g., TK, gE/gI, US3) ensures viral safety while retaining enough replicative fitness to stimulate a strong immune response. Antigen Expression (Middle): Selecting stable promoters (such as EF-1α over CMV) and inserting multiple expression cassettes into non-essential regions enables the creation of highly effective multivalent vaccines (“one shot, multiple diseases”). Immune Enhancement (Right): Co-expressing molecular adjuvants (e.g., IL-18, GM-CSF) helps recruit and activate dendritic cells (DCs), driving a robust cascade that amplifies both innate and adaptive immunity.

In the modern vaccinological paradigm, various viral vector platforms (e.g., mRNA, adenoviral vectors, and poxviral vectors) are undergoing competitive development. Herpesvirus vectors distinguish themselves by successfully circumventing several inherent technical bottlenecks associated with current mainstream platforms. In a comparative analysis, recombinant adenovirus vectors (AdVs) frequently encounter severe interference from pre-existing immunity (neutralizing antibodies) in human populations and specific animal cohorts, which significantly attenuates their immunological efficacy (Awakoaiye et al., 2025; Wang et al., 2022). Conversely, the impact of pre-existing immunity on herpesvirus vectors in heterologous hosts (such as the deployment of avian or porcine viruses as cross-species vectors) is negligible (Gokduman et al., 2025). Furthermore, regarding genomic capacity, the packaging limit of adeno-associated viruses (AAVs) is constrained to approximately 4.7 kb, and that of First-generation AdVs to roughly 7.5–8 kb, severely restricting the expression of complex antigens (Dong et al., 1996; Wold and Toth, 2013). In stark contrast, herpesvirus vectors, typified by pseudorabies virus (PRV, ∼143 kb) and herpesvirus of turkeys (HVT, ∼160 kb), can accommodate exogenous gene expression cassettes of up to tens of kilobases, providing a spatial advantage for the development of multivalent vaccines (Chen et al., 2023; Le Gros et al., 2009; Ye, 2024; Zai et al., 2022). Additionally, compared with mRNA vaccines—which are heavily reliant on stringent cold-chain logistics and exhibit transient antigen expression—herpesvirus vectors demonstrate superior thermal stability (Chaudhary et al., 2021; Liu et al., 2024). Crucially, they can sustain the expression of foreign antigens over extended periods, thereby eliciting robust memory T cell responses that endure for months to years. Consequently, the ultra-large genomic capacity and the durable cellular immune memory mediated by long-term latent infection collectively establish a distinct and robust technological platform for the herpesvirus vector platform (Figure 3 and Table 2). As reverse genetics technologies continue to mature, the specific technical advantages of herpesvirus vectors are manifested in the following domains.

**TABLE 2:** Advantages of herpesvirus vector vaccines compared to other vaccines.

Feature	Herpesvirus vector vaccines	Advantages compared to other vaccines
Genome capacity	Large double-stranded DNA genome (∼150–250 kb)	Can accommodate multiple or large foreign genes, unlike most viral vectors (e.g., adenovirus, poxvirus) with limited capacity.
Host range and tropism	Broad host range and strong tropism for epithelial and immune cells	Enables efficient infection and antigen presentation in various host species and tissues.
Latency and persistence	Ability to establish long-term latent infection without causing disease	Allows sustained antigen expression and long-lasting immunity with fewer boosts.
Immunogenicity	Induces both strong humoral and cellular immune responses	Provides more comprehensive protection than inactivated or subunit vaccines, which mainly induce humoral immunity.
Safety	Non-replicating or replication-deficient vectors are highly attenuated	Safer than conventional, empirically attenuated live vaccines, with low risk of reversion to virulence.
Stability and production	Genetically stable and amenable to large-scale production *in vitro*	Easier to maintain genetic integrity compared to RNA-based vectors.
Multivalent potential	Can carry and express multiple antigens simultaneously	Enables development of polyvalent or multivalent vaccines targeting multiple pathogens or epitopes.
Mucosal immunity	Can stimulate local and systemic immune responses via mucosal routes	Provides advantages over injected subunit or inactivated vaccines that poorly induce mucosal protection.
Use as vaccine platform	Well-established genetic manipulation systems (e.g., BAC technology)	Facilitates rapid design and construction of recombinant vaccines for different diseases.

### 3.1 Safety profiles

Safety is the fundamental prerequisite for the clinical and industrial translation of any novel vaccine vector. The core strategy for modern herpesvirus vectors involves achieving “molecular attenuation” through the targeted deletion of non-essential virulence genes. The herpesvirus genome harbors numerous non-essential genes implicated in pathogenesis and immune evasion, including the thymidine kinase (TK) gene, envelope glycoproteins (e.g., gE and gI), and protein kinases (e.g., US3). Specifically, the deletion of the TK gene significantly restricts viral replication in non-dividing cells (Liu et al., 2024). Studies on the PRV vector SA215 (a TK/gE/gI triple-deletion strain) demonstrated that post-inoculation in swine, the viral DNA load in neurons was reduced by four orders of magnitude (4 log) compared to the wild-type strain, effectively ablating the neurotoxic pathway (Sun L. et al., 2022; Zhu et al., 2011). Similarly, following the knockout of the TK gene in the feline herpesvirus 1 (FHV-1) vector, the recombinant strain FHV-ΔTK exhibited normal proliferation in primary feline kidney cells but was strictly restricted in its replication within neuron-like cells (Lee et al., 2021; Tang et al., 2025; Yang et al., 2023). Furthermore, the ablation of the gE and gI heterodimer not only impairs the trans-synaptic transmission capacity of the virus but also provides a critical marker for DIVA (Differentiating Infected from Vaccinated Animals) strategies. For instance, the classical attenuated PRV Bartha strain, which inherently lacks the gE gene, has been extensively utilized globally as a DIVA vaccine (Delva et al., 2020). Analogously, the deletion of the gE homologous gene in HVT vectors resulted in significantly smaller plaque morphologies in chicken embryo fibroblasts (CEFs) and an inability to disseminate within avian neural tissues, demonstrating an exceptionally high level of biosafety (Boodhoo et al., 2020).

Notably, the targeted deletion of specific antagonistic proteins (such as the PRV US3 kinase and UL41) can abrogate the vector-mediated suppression of host innate immunity, intrinsically conferring a “molecular adjuvant” effect (Yan et al., 2024; Ye et al., 2022). Research indicates that infection of porcine alveolar macrophages (PAMs) with a PRV US3-deletion strain (PRV ΔUS3) induces IFN-β levels 8–10-fold higher than those elicited by the wild-type strain (Bo et al., 2020). In avian models, the deletion of the US3 homolog in Marek’s disease virus type 1 (MDV-1) resulted in enhanced activation of the cGAS-STING pathway in CEFs, subsequently upregulating MHC class I molecule expression by threefold, which significantly elevated antigen presentation efficiency (Li et al., 2025).

In the development of multivalent vaccines, safety considerations logically extend to genetic stability and the risk of virulence reversion (Sun et al., 2023b). To monitor the risk of virulence reversion, the World Organisation for Animal Health (WOAH) mandates that live vectors undergo a test of “at least five back-passages in susceptible animals.” A novel PRV vector maintained stable metrics after five sequential passages *in vivo* in swine, exhibiting no signs of virulence reversion. Predicated on these parameters, vector design must precisely balance the degree of attenuation with replicative fitness: excessive attenuation (e.g., simultaneous deletion of TK, gE, gI, and US9) may lead to a titer reduction of 2 logs, consequently diminishing the protection rate to 60%; conversely, strains retaining TK and exclusively lacking gE/gI can achieve protection rates exceeding 90% (Zheng et al., 2022).

### 3.2 Selection of insertion sites and expression elements: the molecular basis of rational design

The realization of the aforementioned safety profiles and the subsequent optimization of expression are highly contingent upon the rational selection of foreign gene insertion loci and expression elements. The criterion for site determination requires that it must not disrupt critical structural genes while concomitantly evading transcriptional interference. In PRV, loci such as gE, gI, TK, and US9 are frequently selected as insertion sites due to their high permissiveness. In the engineering of HVT, the non-coding intergenic region between UL45 and UL46, as well as the US2 and US10 loci, represent the most robust applications. It is noteworthy that the microenvironment of the insertion site profoundly impacts immunological efficacy. For instance, the insertion of an identical foreign gene into the US2 locus of HVT elicits a significantly more robust specific antibody response than insertion into the US10 locus; this discrepancy may be attributed to the open chromatin conformation and lower transcriptional background interference at the US2 site (Gao et al., 2011). In FHV-1, the deletion of the TK genomic region minimally affects viral *in vitro* replication kinetics, rendering it an optimal “safe harbor” (Tang et al., 2025). Leveraging the capacity of the herpesvirus genome, researchers can meticulously configure multiple non-essential regions to construct multivalent vectors capable of multipathogen protection from a single inoculation. In the poultry sector, the VAXXITEK^®^ series of trivalent vaccines utilizes the HVT vector to express the Newcastle disease virus (NDV) F gene and the infectious bursal disease virus (IBDV) VP2 gene at the US2 and UL45-46 loci, respectively; field trials have validated that a single immunization confers a protection rate exceeding 90% (Le Gros et al., 2009). For felines, the bivalent rFHV-FPV-FCV virus, with antigen genes inserted into the TK and gG loci, respectively, induces titers comparable to those of monovalent vaccines, with no observed competitive antigen inhibition (Tang et al., 2025).

Simultaneously, the meticulous optimization of expression elements is indispensable. The 3’ terminus of the expression cassette must be equipped with a potent transcription termination signal (such as the BGH polyadenylation signal) to effectively preclude RNA polymerase read-through, thereby preventing interference with flanking viral genes. Furthermore, prior to clinical translation, any recombinant virus must undergo rigorous screening via continuous *in vitro* high-passage blind passaging to guarantee genetic stability during large-scale manufacturing.

### 3.3 Optimization strategies for exogenous antigen expression: driving high-efficiency expression

Following the determination of target loci, optimizing the highly efficient and stable expression of exogenous antigens is pivotal for enhancing immunogenicity. Early vectors frequently utilized the human cytomegalovirus (CMV) immediate-early promoter, whose robust transcription initiation capacity drives exceptionally high levels of transient expression during the initial phase. However, its susceptibility to “epigenetic silencing” during persistence *in vivo* has progressively become evident. For example, in a study investigating PRV vector-mediated delivery of CMV-driven EGFP, the fluorescent signal plummeted by 90% at day 30, a phenomenon intimately correlated with elevated methylation levels of the core CpG islands within the CMV promoter (Cabrera et al., 2022). Consequently, contemporary designs have pivoted toward the application of endogenous eukaryotic promoters (such as the EF-1α promoter), which are stably regulated by basal cellular transcription factors and effectively resist gene silencing. Taking the recombinant PRV expressing the CSFV E2 protein as an example, the EF-1α promoter ensured the maintenance of high-level specific antibodies in swine 60 days post-immunization, whereas the CMV group had attenuated to background levels (Brooks et al., 2004). In FHV-1 vectors, the duration of neutralizing antibodies induced by EF-1α-driven expression was prolonged twofold compared to the CMV group (Cabrera et al., 2022; Tang et al., 2025).

Further refined strategies have introduced tissue-specific promoters to achieve substantial increases in expression magnitude. The use of tissue-specific promoters can limit the expression of transgenes in non-target tissues and help ensure sustained expression of the transgenes (Zheng and Baum, 2008). Additionally, in multi-gene designs, deploying a heterologous combination of EF-1α and Pec promoters can circumvent transcriptional competition inherent to homologous promoters, thereby improving the synergistic effect of antigen expression.

### 3.4 Effective enhancement of host immune responses

Herpesvirus vectors have now evolved into highly potent “active immunomodulators.” To overcome immune tolerance, modern designs frequently co-express specific cytokines (such as IL-18 and GM-CSF) as molecular adjuvants alongside target antigens. For example, the sustained secretion of GM-CSF can efficiently recruit immature dendritic cells to the injection site and significantly promote their maturation; concurrently, IL-18 can direct the polarization of naïve T cells toward a Th1-type immune response (Giri and Batra, 2025; van der Heide et al., 2025; Zhu et al., 2024).

Crucially, the underlying mechanistic logic of herpesvirus-induced long-lasting immune protection lies in its unique latency and reactivation cycle. Following the infection of target cells by the recombinant virus, its DNA genome persists stably within the host cell nucleus as a circular episome. During latency, the virus actively represses the expression of its own lytic genes via the high-level transcription of latency-associated transcripts (LATs), thereby elegantly evading complete clearance by the host immune system. This “stealth” persistence ensures the long-term, “trickle-down” release of foreign antigens within the host. This continuous, low-level antigenic stimulation, coupled with the aforementioned type I IFN inflammatory microenvironment induced via the cGAS-STING pathway, enables the highly efficient cross-presentation of target antigens via the MHC class I pathway. This provides sustained activation signals to memory B cells and effector/memory CD8+ T cells (CTLs). *In vivo* experiments have demonstrated that post-inoculation with such vector vaccines, neutralizing antibodies and key cytokines such as IL-2 and IFN-γ are maintained at high levels for prolonged periods (van der Heide et al., 2025).

In summary, armed with distinct molecular biological characteristics and the advantage of vast genomic capacity, herpesvirus vectors demonstrate substantial comprehensive competitiveness in the field of vaccine design. Precise deletion of virulence genes concomitantly confers safety and intrinsic adjuvant effects; rational optimization of insertion loci and expression elements ensures genetic stability; the implementation of diversified promoter strategies facilitates high-efficiency antigen expression; and reliance on episomal latency mechanisms elicits exceptionally durable cellular and humoral immunity. Under the “One Health” framework, multivalent herpesvirus vector vaccines not only streamline immunization protocols and alleviate physiological stress but also effectively sever the transmission chains of zoonotic diseases. Propelled by continuous innovations in reverse genetics and precision editing technologies, herpesvirus vectors are well-positioned to poised to serve as a robust foundational platform for the development of next-generation recombinant vaccines.

## 4 Applications and current landscape of animal herpesvirus vector development

**FIGURE 4:**
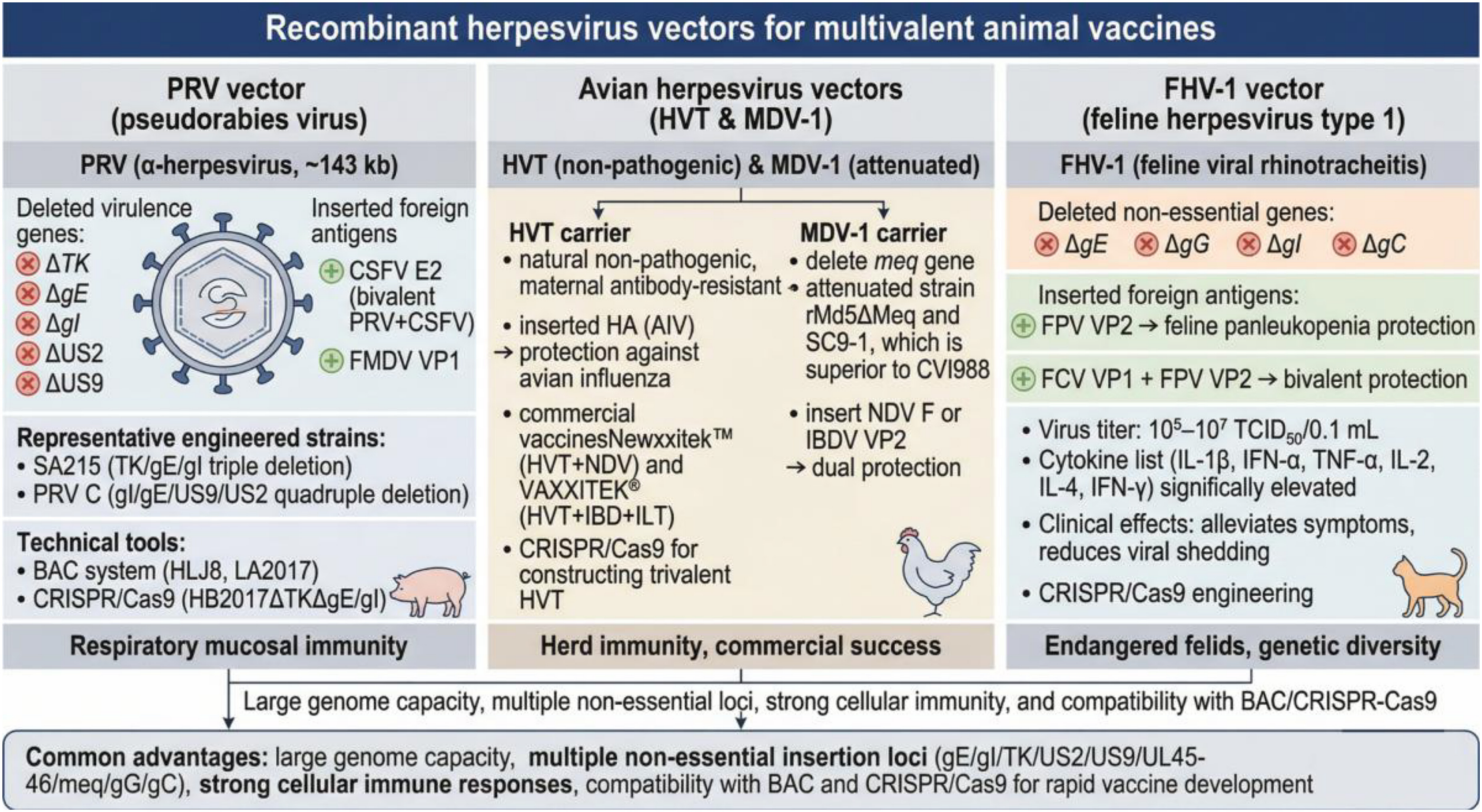
Applications of recombinant herpesvirus vectors for multivalent animal vaccines. PRV vectors (Left): Engineered for swine by deleting specific virulence genes (e.g., TK, gE, gI) to express foreign antigens like CSFV E2, effectively stimulating respiratory mucosal immunity. Avian herpesvirus vectors (Middle): Utilizes non-pathogenic HVT or attenuated MDV-1 (e.g., meq-deleted strains) to deliver antigens against major poultry diseases, achieving robust herd immunity and broad commercial success. FHV-1 vectors (Right): Designed for felines by deleting non-essential genes to express viral antigens (e.g., FPV VP2, FCV VP1), providing bivalent protection and helping preserve the genetic diversity of endangered felids.

Animal herpesvirus vectors occupy a pivotal strategic position in the research and development (R&D) of contemporary veterinary biological products. The substantial capacity of their large genomes for accommodating exogenous genes, coupled with their inherent ability to induce durable immunity, establishes them as an ideal vector platform for the construction of multivalent vaccines. The simultaneous expression of multiple protective antigens from diverse heterologous pathogens within a single viral backbone profoundly streamlines immunization protocols and confers broad-spectrum protection. This section provides an in-depth examination of the current applications and developmental landscape of porcine pseudorabies virus (PRV), avian Marek’s disease virus (MDV) and the herpesvirus of turkeys (HVT), as well as feline herpesvirus type 1 (FHV-1) (Figure 4 and Table 3).

**TABLE 3:** Common insertion sites and related recombination information of herpesvirus vectors.

Vector virus	Insertion site	Promoter	Termination sequence	Exogenous gene	References
PRV	PK-gG TK, gE, gI, and UL39	CMV	SV40	PPV1 -VP2	(Tian et al., 2025)
	gE/gI/TK	CMV	SV40	PEDV-S1	(Jiao et al., 2024)
	gE/gI/TK	gG (PRV)	SV40	HP-PRRSV-GP5	(Zheng et al., 2025)
	US4-US6	CMV	SV40	CSFV-E2	(Sun et al., 2023a)
HVT	UL3-4	Chicken β-actin promoter	PolyA	IBDV-VP2	(Andoh et al., 2017)
	UL22-23	Chicken β-actin promoter	PolyA	IBDV-VP2	(Andoh et al., 2017)
	UL45-46	Chicken β-actin promoter	PolyA	IBDV-VP2	(Andoh et al., 2017)
		CMV enhancer and chicken β-actin chimeric promoter	SV40	H7N9 subtype AIV-HA	(Chen et al., 2023)
		EF-1α	BGH	H7N1 subtype AIV-HA	(Chen et al., 2023)
	US10-SORF3	Chicken β-actin promoter	PolyA	IBDV-VP2	(Zhang et al., 2024)
	US1-10	CMV	BGH	NDV-F	(Shi et al., 2024)
	US2	LTR	SV40	ILTV-gD	(Shao et al., 2025)
MDV	UL55	Chicken β-actin promoter	PolyA	NDV-F	(Wu et al., 2017)
	UL41, US2, US10	Chicken β-actin promoter	PolyA	IBDV-VP2	(Li et al., 2016)
	US2	Chicken β-actin promoter	PolyA	H9N2-HA	(Chen et al., 2024)
FHV-1	TK	CMV	SV40	FeLV env, gag, gp70, gp85/FCV capsid/FPV VP2	(Tang et al., 2025)
	UL	CMV	SV40	FIV env, gap	(Sato et al., 2000)

### 4.1 Pseudorabies virus (PRV) vectors

Pseudorabies virus (PRV) serves as a paramount animal model in molecular biology, characterized by a genome of approximately 143 kb. This genome harbors multiple non-essential virulence genes (e.g., TK, gE, gI, US2, and US9) that dictate neurotropism and pathogenicity. The precise deletion of these loci constitutes the fundamental strategy for engineering attenuated backbones, enabling the generation of intrinsically safe strains that exhibit restricted replication in non-dividing cells and an abrogation of trans-synaptic transmission capabilities, while concurrently preserving replicative fitness. Representative attenuated strains, such as SA215 (a TK/gE/gI triple-deletion mutant) and the PRV C strain (a gI/gE/US9/US2 quadruple-deletion mutant), have demonstrated exemplary safety profiles and exceptional immunogenicity in field trials (Sun L. et al., 2022; Zhu et al., 2011).

In the context of multivalent vaccine development, the primary advantage of PRV vectors resides in their capacity to simultaneously express multiple antigens from major infectious pathogens within a single vector system. Exploiting genomic deletion sites, researchers have successfully inserted the E2 gene, a critical protective antigen of the classical swine fever virus (CSFV), generating a bivalent PRV-CSFV vaccine capable of simultaneously conferring protection against both pseudorabies and classical swine fever in piglets following challenge (Sun et al., 2023a). Similarly, the integration of the foot-and-mouth disease virus (FMDV) VP1 gene has facilitated the successful development of a recombinant bivalent vaccine (Qian et al., 2004). In response to the emergence of novel variant strains, an attenuated mutant, HB2017ΔTKΔgE/gI, was rapidly developed based on the circulating variant (Ling et al., 2025; Tian et al., 2025). This mutant features an extensive modification capacity, functioning as a universal platform capable of accommodating multiple independent expression cassettes. The establishment of bacterial artificial chromosome (BAC) systems and CRISPR/Cas9-mediated scarless construction techniques have dramatically accelerated the R&D cycle. Globally, several PRV vector vaccines have advanced to clinical trials or achieved commercialization. Nevertheless, Nevertheless, to circumvent the bottlenecks of promoter competition and transcriptional interference inherent in the co-expression of multiple heterologous proteins, the multivalent design of modern herpesvirus vectors, such as pseudorabies virus (PRV), relies heavily on precise molecular engineering strategies. First, the deployment of heterologous promoter combinations is critical to alleviate transcription factor competition; substituting tandem homologous strong promoters (e.g., dual CMV promoters) with a heterologous array—such as pairing a ubiquitous endogenous promoter (e.g., EF-1α) with a tissue-specific promoter (e.g., Pec)—effectively prevents the exhaustive depletion of the shared transcription factor pool within the target cell. Second, optimizing the spatial separation and transcriptional orientation of expression cassettes is equally paramount. Independent cassettes are typically distributed across distant, non-essential genomic loci (e.g., sequentially targeted to the gE/gI and TK loci); if multiple cassettes must be inserted in close proximity, a divergent (head-to-head or back-to-back) transcriptional orientation is utilized to significantly minimize RNA polymerase collisions and steric hindrance during elongation. Finally, the incorporation of robust bidirectional polyadenylation signals (e.g., the BGH polyA signal) at the 3’ terminus of each cassette serves as a potent transcription termination element, strictly precluding RNA polymerase read-through that might otherwise epigenetically silence adjacent downstream genes. Through these sophisticated three-dimensional molecular configurations, recombinant herpesvirus vectors effectively bypass transcriptional bottlenecks during multigene co-expression, thereby firmly validating the feasibility and stability of platforms like PRV as robust, multivalent vaccine vehicles.

### 4.2 Avian herpesvirus vectors: MDV and HVT

The herpesvirus of turkeys (HVT) and Marek’s disease virus type 1 (MDV-1) represent the most prominent examples of commercial application for recombinant viral vectors. The most salient advantage of HVT is its inherent non-pathogenicity in poultry, enabling it to effectively circumvent the interference of maternally derived antibodies. Following inoculation, HVT establishes a lifelong latent infection within the lymphoid compartment of the avian host, thereby ensuring the continuous and sustained delivery of antigens. Conversely, for the pathogenic MDV-1, the targeted deletion of the oncogene meq (exemplified by the rMd5 ΔMeq and SC9-1 attenuated strains) markedly reduces its tumorigenic properties, yielding a protective efficacy that demonstrates superior protective potential against highly virulent MDV strains compared to CVI988 in experimental settings that of the conventional CVI988 live vaccine (Su et al., 2015; Sun et al., 2020).

Multivalent recombinant HVT vaccines have fundamentally revolutionized poultry immunization protocols. Commercial vaccines utilizing the HVT backbone to express the Newcastle disease virus (NDV) F gene confer robust, dual protection against both Marek’s disease and Newcastle disease following a single hatchery administration. Moreover, HVT vectors expressing the avian influenza virus (AIV) HA gene establish a highly robust herd immunity barrier, markedly mitigating the risk of AIV spillover into mammalian populations. Similarly, MDV-1 vectors expressing the NDV F protein or IBDV VP2 protein have been empirically validated to provide potent dual protection (Shah et al., 2022). The advent of CRISPR/Cas9 technology has significantly streamlined the screening of recombinants harboring multiple antigens. The deployment of these multivalent vaccines drastically reduces the frequency of inoculations and associated physiological stress, seamlessly aligning with the pragmatic requirements of intensive husbandry. Globally, the technological maturity of HVT-based recombinant vaccines is exceedingly high; future trajectories will likely prioritize the incorporation of tissue-specific promoters and the rapid iteration of updated vaccine candidates.

### 4.3 Feline herpesvirus type 1 (FHV-1) vectors

The genome of feline herpesvirus type 1 (FHV-1) comprises multiple glycoprotein genes (e.g., gE, gG, gI, and gC) that are dispensable for replication *in vitro*. The targeted deletion of these genomic loci significantly attenuates viral pathogenicity toward the feline upper respiratory mucosa without compromising *in vitro* proliferation capabilities. Notably, the TK genetic locus—owing to its minimal impact on viral growth following deletion and its provision of ample spatial capacity—has been established as the optimal “safe harbor” for exogenous gene insertion.

FHV-1 exhibits translational potential for the multiplexed prophylaxis of companion animals. Utilizing an attenuated FHV-1 backbone to insert the VP2 gene of feline panleukopenia virus (FPV), researchers have constructed a bivalent recombinant vaccine that effectively prevents both feline viral rhinotracheitis and panleukopenia (Tian et al., 2025). Furthermore, a trivalent vaccine candidate co-expressing the feline calicivirus (FCV) VP1 and FPV VP2 proteins signifies a critical advancement toward highly integrated vector vaccines for companion animals (Tian et al., 2025). These recombinant viruses demonstrate stable proliferation in susceptible cell lines, fulfilling the criteria for commercial-scale manufacturing, and maintain genetic stability across successive passages. Mechanistic immunological studies indicate that these vectors not only elicit high-titer neutralizing antibodies but also induce significantly elevated transcriptional levels of critical cytokines, such as IL-1β and IFN-α, in peripheral blood mononuclear cells (PBMCs), thereby substantially ameliorating clinical manifestations and viral shedding post-challenge (Tang et al., 2024). Concurrently, FHV-1 vectors are being investigated for the conservation of critically endangered felids, such as the snow leopard (Wu et al., 2022). However, the high prevalence of pre-existing FHV-1 antibodies in adult feline populations presents a substantial risk of primary neutralization; consequently, future optimization of inoculation routes, prioritizing mucosal immunization, is imperative to circumvent interception by circulating antibodies.

### 4.4 Comparative analysis of animal herpesvirus vectors and the comprehensive advantages of multivalent vaccines

Porcine PRV and avian HVT/MDV possess an abundance of well-characterized non-essential genomic regions, profoundly facilitating the construction of complex, multi-antigen expression cassettes. HVT, leveraging its inherent non-pathogenicity and capacity to overcome maternally derived antibodies, firmly retains a dominant position in commercialization. Conversely, the FHV-1 vector demonstrates an irreplaceable target-specific advantage in the context of companion animal prophylaxis and wildlife ecological conservation. The vast DNA genomes of these three vector classes readily accommodate multiple expression cassettes. When synergized with their natural mechanisms of long-term latency, these platforms are endowed with the capacity to elicit durable, multifaceted immune responses. Furthermore, underpinned by advanced reverse genetics technologies, these systems can rapidly respond to emerging variant strains with unparalleled efficiency. By substantially minimizing immunization-induced stress and injection frequency, these multivalent platforms significantly elevate the biosecurity paradigms of modern animal husbandry and companion animal care. Ultimately, owing to their massive carrying capacity, robust safety profiles, and intrinsic ability to stimulate persistent immunity, porcine, avian, and feline herpesvirus vectors occupy an indispensable role in the rational design of multivalent vaccines. As the field progresses, the next generation of herpesvirus vectors will unequivocally advance toward heightened precision, efficacy, and broad-spectrum capability, thereby constructing a formidable immunological barrier for the global eradication of animal diseases.

## 5 Future perspectives and challenges of recombinant herpesvirus vaccines

### 5.1 Comprehensive applications within the “one health” framework

Recombinant herpesvirus vector vaccines represent a profound biomedical innovation, offering distinct advantages within the “One Health” paradigm. By integrating animal health, human health, and environmental stewardship, this holistic approach provides novel strategies for the control of emerging and re-emerging infectious diseases. In the context of swine health management, PRV-based vector vaccines not only confer protection against pseudorabies but also serve as a versatile platform for multivalent vaccine development. Studies have demonstrated that the incorporation of antigens from zoonotic pathogens, such as the swine influenza virus, into PRV vectors can significantly mitigate the risk of cross-species transmission (Petro-Turnquist et al., 2024). Such strategies are particularly valuable for establishing robust biosecurity barriers and severing the transmission chains of zoonotic diseases. Within the poultry sector, HVT and MDV-1 vaccines not only safeguard avian health but also establish herd immunity, thereby drastically reducing the risk of avian influenza spillover into human populations (Kamel and El-Sayed, 2019; Kamel et al., 2023; Liu et al., 2022). Beyond zoonotic spillover, herpesvirus vectors also contribute to the ecological and environmental pillar of the One Health framework. Advancements in FHV-1-based vectors targeting felines and endangered species (e.g., snow leopards) provide critical tools for disease management in susceptible wild populations. Rather than zoonotic control, these applications fundamentally support ecological conservation, helping to maintain ecological balance and preserve genetic biodiversity in endangered species (Kamel and El-Sayed, 2019; Yu et al., 2023). Furthermore, these vector systems also offer crucial translational insights for human medicine; for instance, MDV-mediated tumor antigen presentation has informed the development of vaccines against Epstein-Barr virus (EBV)-associated malignancies (Naskar et al., 2024). Consequently, interdisciplinary technological exchange is increasingly accelerating vaccine advancement.

### 5.2 Key technical considerations in vaccine design

The successful design of these vaccines necessitates the integration of multiple technical parameters. Antigen selection must balance immunogenicity with evolutionary conservation, and the choice of appropriate antigen formats (e.g., full-length proteins, functional domains, or multi-epitope fusions) exerts a decisive impact on vaccine efficacy. When engineering complex multi-epitope fusion constructs within the expansive herpesvirus genome, empirical sequence selection is no longer sufficient. Modern vaccinology increasingly relies on the integration of advanced computational structural prediction tools, such as AlphaFold 3, into the rational design pipeline. This structural validation is imperative to ensure that chimeric proteins fold correctly without steric hindrance and present stable, highly neutralizing pre-fusion epitopes. By leveraging these predictive structural models prior to *in vitro* construction, researchers can prevent the misfolding of multivalent antigens, thereby maximizing the immunogenic potential and targeted efficacy of the recombinant vectors. Vector optimization remains a core component: PRV is highly amenable to respiratory mucosal immunization (with stable expression at loci such as gE/gI and US9), whereas avian herpesvirus insertion sites, including UL45-46 and US2, facilitate high-level expression. Concurrently, the design of the expression cassette significantly dictates overall vaccine performance. The utilization of tissue-specific promoters (e.g., the Pec promoter) has been shown to enhance antigen expression by up to fourfold compared to ubiquitous promoters such as CMV (Zheng and Baum, 2008). Furthermore, the selection of insertion sites requires a comprehensive evaluation of the local genomic microenvironment; for instance, the US2 locus in HVT induces superior antibody responses due to enhanced antigen presentation efficiency (Gao et al., 2011), while the deletion of the TK locus in FHV-1 minimally impacts viral replication while permitting robust transgene expression (Tian et al., 2025).

### 5.3 Current challenges, clinical translation, and regulatory considerations

Despite their highly promising prospects, the comprehensive industrialization of herpesvirus vector vaccines continues to encounter dual challenges in manufacturing processes and regulatory approval. Firstly, recombinant herpesviruses exploit episome-mediated latent infection to provide sustained, low-dose antigenic stimulation to the host, the potential risk of spontaneous reactivation and subsequent environmental shedding of the live vector under conditions of physiological stress or immunosuppression constitutes a non-negligible biosafety challenge. Under high-stress scenarios—such as intensive livestock and poultry farming, high-density transportation, or environmental changes—the recombinant vector, initially in a latent state, may be reactivated to resume lytic replication. This not only leads to unintended horizontal transmission of the pathogen within susceptible populations, but more critically, the shed vector following reactivation is highly prone to homologous recombination with naturally circulating wild-type field strains or pre-existing latent strains within the host, thereby posing the risk of generating novel recombinant variant strains. To achieve an optimal rational balance between clinical immune durability and environmental biosafety, next-generation vector engineering is currently focusing on severing the reactivation pathway at the molecular level. Prominent strategies include developing “latency-deficient” vectors; Alternatively, researchers are constructing “replication-defective” vector systems that completely lack autonomous replicative capacity *in vivo*. These designs aim to preserve the vast cargo capacity and robust initial immunogenicity of herpesviruses while completely abrogating the risks of post-latency reactivation and environmental shedding, thereby mitigating potential ecological hazards following environmental release and satisfying the stringent regulatory safety standards for modern biological products.

Regarding clinical translation and manufacturing processes, large-scale vaccine production is constrained by technical bottlenecks pertaining to titer maintenance and cost optimization. Mechanistically, although herpesvirus vectors possess massive genomes, the tandem insertion of multiple genes inevitably compromises the replicative fitness of the virus. For example, the insertion of more than three independent, large exogenous expression cassettes into a PRV vector can result in a significant titer reduction of 1–2 logs during cell culture (Yang et al., 2024). Furthermore, transitioning from conventional adherent cell cultures to high-density suspension culture processes introduces substantial difficulties in maintaining the stable proliferation of recombinant multivalent viruses. Overcoming the hurdles associated with downstream purification and high-recovery concentration of large, enveloped viruses in large-scale bioreactors represents a core manufacturing challenge that restricts clinical translation.

From a regulatory perspective, multivalent live vaccines based on recombinant herpesviruses face exceedingly stringent approval thresholds from agencies such as the US Food and Drug Administration (FDA), the European Medicines Agency (EMA), and the World Organisation for Animal Health (WOAH). Regulatory authorities are acutely focused on the risks associated with the “environmental release” of such genetically engineered live vaccines. A primary concern is whether live vectors harboring core antigens from multiple pathogens might engage in unpredictable horizontal gene transfer within complex field microbiomes. Moreover, the continuous monitoring of virulence reversion risks constitutes a critical evaluative metric. During the regulatory submission process, manufacturers must provide exhaustive data packages encompassing, but not limited to: deep sequencing analyses of genetic stability across continuous *in vitro* and *in vivo* passages (typically spanning dozens of generations); precise evaluations of *in vivo* biodistribution; and longitudinal studies detailing the shedding and transmission kinetics within target animal populations.

**FIGURE 5:**
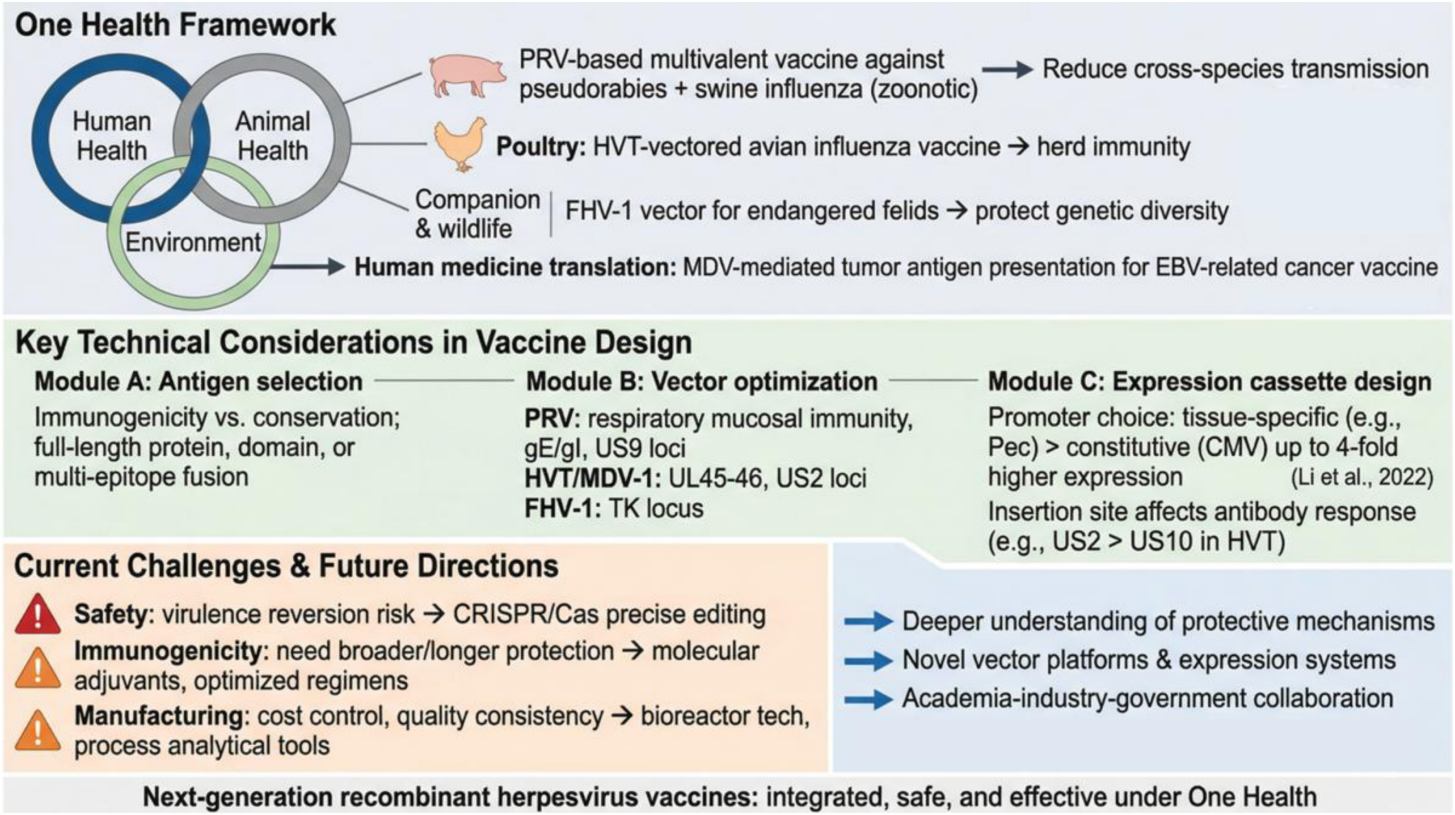
Comprehensive overview of recombinant herpesvirus vaccines under the One Health framework. One Health Applications (Top): Illustrates the cross-species utility of viral vectors (PRV, HVT, FHV-1) in swine, poultry, and wildlife to reduce zoonotic transmission and protect animal health, alongside their translational potential for human medicine (e.g., cancer vaccines). Technical Considerations (Middle): Highlights key design modules, including optimal antigen selection, specific vector loci modification (e.g., TK, gE/gI, US2), and rational expression cassette design (promoter and insertion site choices). Challenges and Future Directions (Bottom): Outlines current hurdles in vaccine safety, immunogenicity, and manufacturing, while pointing toward future solutions such as precise CRISPR/Cas editing, novel vector platforms, and multi-sector collaboration.

Despite these formidable manufacturing and regulatory barriers, such challenges are not insurmountable. The implementation of modern process analytical technology (PAT) is progressively addressing quality consistency issues during the scale-up of suspension cultures. Concurrently, precise and scarless CRISPR-mediated repair strategies are fundamentally mitigating the risks of recombinant escape mutations at their source. Future research endeavors should continue to elucidate the underlying immunological mechanisms through which recombinant vectors confer protection, while developing novel vector platforms to drive technological innovation. Fostering profound mutual trust and collaboration among academia, industry, and governmental regulatory bodies will be essential to accelerate the establishment of robust evaluation frameworks, ultimately ensuring that these pioneering next-generation recombinant vaccines can be introduced to the market with safety and efficacy (Figure 5).

## 6 Conclusion

In conclusion, recombinant animal herpesviruses represent a paradigm-shifting platform in modern vaccinology, successfully bridging the gap between basic reverse genetics and pragmatic clinical applications. By leveraging their substantial genomic payload capacity and unique host-interaction mechanisms, these vectors facilitate the development of multivalent vaccines that confer robust, long-lasting cellular and humoral immunity from a single inoculation, while effectively circumventing the interference of pre-existing immunity commonly observed with other viral vectors. The transition toward high-precision genome editing technologies has further cemented their safety profiles by enabling stable, scarless molecular attenuation and intrinsic adjuvant effects.

Nevertheless, the realization of their full commercial and translational potential necessitates overcoming substantial technical and regulatory barriers. Future developmental trajectories must prioritize the optimization of large-scale, high-density suspension manufacturing processes, ensuring the replicative fitness and genetic stability of highly multiplexed vectors. Concurrently, navigating the stringent regulatory requirements demands comprehensive, longitudinal data regarding *in vivo* biodistribution, virulence reversion risks, and environmental shedding. Looking forward, strategically integrated under the “One Health” initiative, the continuous refinement of rationally designed multivalent herpesvirus vaccines is unequivocally poised to enhance global biosecurity, providing an indispensable, broad-spectrum arsenal for the eradication of complex veterinary and zoonotic pathogens.
